# The role of copeptin as a diagnostic and prognostic biomarker for risk stratification in the emergency department

**DOI:** 10.1186/1741-7015-10-7

**Published:** 2012-01-20

**Authors:** Christian H Nickel, Roland Bingisser, Nils G Morgenthaler

**Affiliations:** 1 Emergency Department, University Hospital, Basel, Switzerland; 2 Institut für Experimentelle Endokrinologie und Endokrinologisches Forschungszentrum, EnForCé, Charité, Campus Virchow, Berlin, Germany

## Abstract

The hypothalamic-pituitary-adrenal axis is activated in response to stress. One of the activated hypothalamic hormones is arginine vasopressin, a hormone involved in hemodynamics and osmoregulation. Copeptin, the C-terminal part of the arginine vasopressin precursor peptide, is a sensitive and stable surrogate marker for arginine vasopressin release. Measurement of copeptin levels has been shown to be useful in a variety of clinical scenarios, particularly as a prognostic marker in patients with acute diseases such as lower respiratory tract infection, heart disease and stroke. The measurement of copeptin levels may provide crucial information for risk stratification in a variety of clinical situations. As such, the emergency department appears to be the ideal setting for its potential use. This review summarizes the recent progress towards determining the prognostic and diagnostic value of copeptin in the emergency department.

## Introduction

Risk stratification is a core task in emergency medicine. Novel biomarkers such as pro-calcitonin and copeptin have emerged to assist clinicians with decision-making. Elevated pro-calcitonin levels, for example, might help to decide whether administration of antibiotics in lower respiratory tract infections is necessary [1,2].

**Figure 1:**
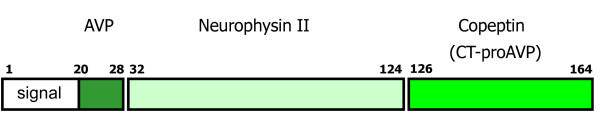
**Cartoon of the 164-amino acid peptide precursor, preprovasopressin**. Shows the signal sequence (white), AVP (dark green), neurophysin II (pale green) and copeptin (light green). Copeptin (CT-proAVP) is the C-terminal part of proAVP. Numbers indicate amino acids of the human protein. AVP: arginine vasopressin; CT-proAVP: C-terminal proAVP; signal: signal peptide.

**Figure 2:**
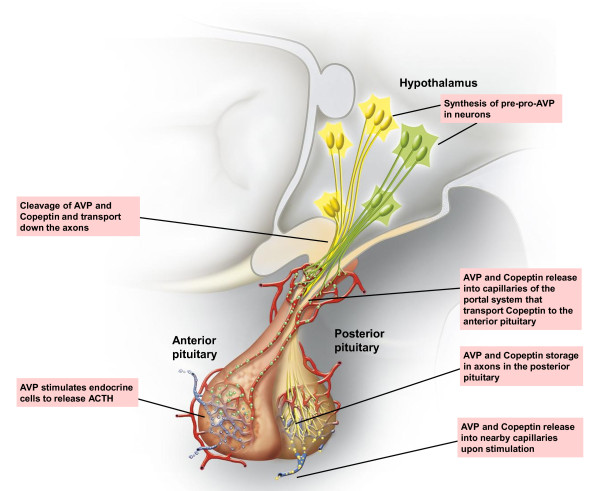
**Synthesis and release of AVP and copeptin in hypothalamus and pituitary**. Pro-AVP is processed in the hypothalamus, followed by two distinct release-mechanisms for the anterior and posterior pituitary. During stress, a drop in blood pressure, or a change in osmotic pressure, AVP is released into the circulation. ACTH: adrenocorticotropic hormone; AVP: arginine vasopressin.

Arginine vasopressin (AVP), also known as antidiuretic hormone, is one of the key hormones of the hypothalamic-pituitary-adrenal (HPA) axis. Copeptin, a peptide of 39 amino acids, is the C-terminal part of pro-AVP and is released together with AVP during processing of the precursor peptide (see Figure 1) [3]. Copeptin and AVP are secreted from the neurohypophysis upon hemodynamic or osmotic stimuli (see Figure 2). AVP is also involved in the endocrine stress response. Corticotropin-releasing hormone and AVP appear to have a synergistic effect, resulting in adrenocorticotropic hormone (ACTH) and cortisol release [4-7]. High cortisol levels reflect a higher degree of stress, but are dependent on the integrity of the HPA-axis [8,9]. Copeptin appears to be superior to cortisol in determination of the stress level, as cortisol is further downstream in the stress response, has a strong circadian rhythm and is also challenging to measure as a free hormone [10].

In contrast to AVP and cortisol, copeptin is stable both in serum and plasma at room temperature and can be easily measured *ex vivo *as a 'shadow' fragment of AVP in the circulation [11,12], in manual or fully automated chemiluminescence assays. Copeptin results are available within one hour, which is crucial for any useful biomarker in the emergency department (ED) setting.

This mini review outlines the potential prognostic and diagnostic use of copeptin in the ED in the context of a number of different possible clinical conditions and summarizes the recent progress made in this field.

### Effects of copeptin and AVP in the circulation

The physiologic function of AVP is threefold. When released into the circulation, AVP mediates arteriolar vasoconstriction via the V_1_-receptor and exhibits an antidiuretic effect in the kidneys via the V_2_-receptor [13]. A third AVP receptor appears to be involved in the secretion of ACTH [14]. At present, it is unclear if copeptin has a physiologic effect outside the neuron, where it acts as a chaperone in the maturation process of AVP. It is noteworthy that, *in vivo*, the kinetics of copeptin are similar to those of AVP [12,15], whilst, *ex vivo*, the protein has an extraordinary stability of one to two weeks at room temperature [12]. This favorable discrepancy allows for the precise measurement of copeptin as a surrogate marker for the unstable AVP.

### Copeptin in acute myocardial infarction and heart failure

Early and safe rule-out of myocardial infarction (MI) is crucial in the ED. Reichlin *et al*. examined the role of copeptin in the diagnosis of 487 consecutive patients with chest pain presenting to the ED [16]. Copeptin levels were already elevated at a time when troponin T was still undetectable (0 hours to 4 hours) in 20 out of 81 patients with the final diagnosis of acute MI. In their study, negative troponin and copeptin at the time of ED presentation was enough to rule out acute MI, with an excellent negative predictive value of 99.7% [16]. This was confirmed in a similar study by Keller *et al*. [17]. However, recent studies have shown an increased diagnostic sensitivity of high-sensitivity troponin assays compared to conventional assays in the early detection of acute MI [18,19].

At present, there is confusion on the nomenclature and clinical use of these assays [20]. This needs to be clarified before the benefit of additional biomarkers can be fairly evaluated. First reports on a direct comparison of high-sensitivity troponin assays and copeptin confirm the additive effect [21], and the results of the ongoing CHOPIN trial (Copeptin Helps in the early detection Of Patients with acute myocardial INfarction), a multicenter trial of 2000 patients with chest pain in the ED, should answer many of the open questions (ClinicalTrials.gov Identifier: NCT00952744).

In several studies, copeptin has been reported to be a useful biomarker in patients with chronic heart failure. High levels of copeptin predicted poor long-term prognosis in patients with chronic heart failure [22,23]. When combined with B-type natriuretic peptide (BNP) measurement, outcome prediction could be improved further [22,23].

When copeptin was measured in 980 patients who had experienced an acute MI, it was found to be elevated in patients who died or were readmitted with heart failure, compared with survivors. Copeptin and N-terminal proBNP were independent predictors of death or heart failure at 60 days, helping to stratify patients into low-, intermediate- or high-risk groups [24]. In a multicenter study, it was confirmed that copeptin is a strong marker for mortality and morbidity in patients with heart failure after an acute MI [25].

The clinical evidence in this field is increasing rapidly. A recent study of patients treated in an outpatient clinic for chronic stable heart failure showed that copeptin and high-sensitivity cardiac troponin T (hs-cTnT) levels, both single and combined, are powerful predictors of death and hospitalization. This suggests that simultaneous assessment of myocardial damage and the activated vasopressin system, through single measurement of hs-cTnT and copeptin levels, might be of prognostic relevance [26].

Copeptin may have not only short term prognostic relevance, but also identify patients at higher long-term risk. Increased copeptin concentrations in elderly patients with symptoms of heart failure were associated with an increased risk of all-cause mortality after a lengthy median follow-up of 13 years [27].

Finally, a secondary analysis from the Biomarkers in Acute Heart Failure (BACH) study looked at the interaction of copeptin and sodium concentrations. Whilst it is well known that low serum sodium is an indicator of increased mortality in patients with heart failure, no studies examined if and how the vasopressinergic system was activated. The authors of the BACH study now report significantly increased 90-day mortality, readmissions and ED visits in patients with hyponatremia, especially in those with elevated copeptin. Patients with hyponatremia and low copeptin had a lower risk. Copeptin was highly prognostic for 90-day adverse events in acute heart failure patients, adding prognostic value to clinical predictors, serum sodium and natriuretic peptides. This report is of particular interest in the light of ongoing discussions on the potential value of vasopressin receptor antagonists (such as tolvaptan) for patients with heart failure, as elevated copeptin may identify those patients with an activated AVP system most likely to benefit from AVP antagonists [28].

### Copeptin in hyponatremia and diabetes insipidus

Hyponatremia is common in the ED, but the diagnostic approach is often challenging [29]. In the differential diagnosis of the syndrome of inappropriate antidiuretic hormone hypersecretion and sodium depletion, copeptin showed promising results [30]. However, a large overlap in copeptin levels still makes the differentiation between these two disorders challenging. In this context, the copeptin to urinary sodium ratio was most helpful. Further studies are needed here and currently ongoing. In other situations of disturbed water balance, such as diabetes insipidus, the role of copeptin was also investigated. Three studies showed promising results of copeptin in the differential diagnosis of polydipsia-polyuria syndrome [30-32]. However, according to a recent study, the value of copeptin might be restricted to patients with no further acute illness, such as stroke or pneumonia, besides a sodium imbalance [33].

### Copeptin as a prognostic marker

Besides the clinical indications mentioned so far, the role of copeptin as a prognostic biomarker was also examined in a variety of other indications, such as acute exacerbations of chronic obstructive pulmonary disease [34], lower respiratory tract infections [35], hemorrhagic and septic shock [15], stroke [36,37] and traumatic brain injury [38]. Table 1 summarizes the recent study results.

**Table 1:** Overview of studies investigating the use of copeptin in different clinical settings.

Clinical scenario	Setting	Number of patients	Outcome
AECOPD [34]	ED	167	Copeptin levels > 40 pmol/L associated with prolonged hospital stay and long-term clinical failure (death or rehospitalization for AECOPD up to six months after inclusion).

LRTI [35]	ED	545	Copeptin levels increased with increasing severity of LRTI, as classified by the Pneumonia Severity Index - predictive of mortality (AUC 0.75) - copeptin levels in survivors 24.3 pmol/L (normal: 10.8 to 43.8 pmol/L), versus 70.0 pmol/L (normal: 28.8 to 149 pmol/L) in non-survivors - optimal threshold of copeptin, 53 pmol/L; sensitivity to correctly predict mortality, 58% with a specificity of 80%; LR+ 3.0, LR- 0.5

Hemorrhagic and septic shock [15]	ICU	101	Copeptin levels increased with disease severity from systemic inflammatory response syndrome to sepsis and severe sepsis to septic shock - copeptin levels in non-survivors, 171.5 pmol/L versus survivors, 86.8 pmol/L, *P *< 0.001 - predictive of mortality (AUC 0.75) - optimal prognostic accuracy of copeptin at 96 pmol/L; sensitivity, 61.5%, specificity 83.8%

Acute ischemic stroke [49]	ED	362	Copeptin - associated with severity of stroke and lesion size - predicts functional outcome (AUC 0.73) and mortality (AUC 0.82) after three months

Cerebrovascular re-event after transient ischemic attack within 90 days [36]	ED	107	- AUC for copeptin to predict re-event within 90 days, 0.73. - at cutoff of 9.0 pmol/L for copeptin; sensitivity, 80%, specificity, 76%

One-year outcome in patients with acute stroke [37]	One-year follow-up on ED patients	341 of 362	Copeptin predicts - one-year mortality after stroke (AUC 0.74) - functional outcome (AUC 0.72)

Acute spontaneous intracerebral hemorrhage [50]	ED	40	Copeptin predicts - 30-day mortality (AUC 0.88), comparable with that of GCS (AUC 0.82) - 90-day functional outcome (AUC 0.68)

Traumatic brain injury [38]	Neurosurgery	94	Copeptin - predicts one-month mortality (AUC 0.874), similar to that of GCS - copeptin levels > 451.8 pg/mL; sensitivity, 88.5%, specificity, 75%, in prediction of one-month mortality

AECOPD: acute exacerbation of chronic obstructive pulmonary disease; AUC: area under the curve; ED: emergency department; GCS: Glasgow Coma Scale; ICU: intensive care unit; LR-: negative likelihood ratio; LR+: positive likelihood ratio.

### Copeptin in elderly ED patients presenting with non-specific complaints

Older patients in the ED are at risk of adverse health outcomes [39]. Atypical disease presentation or presentation with non-specific complaints, such as weakness or fatigue, is common [40]. The attenuated physiologic reaction of elderly patients in response to even serious illness complicates the evaluation even further. The Basel non-specific complaints (BANC) study aims to evaluate risk-stratification tools for these, mostly elderly, patients presenting to the ED with non-specific complaints [40]. In this study, copeptin concentrations were significantly higher in non-survivors than in survivors, irrespective of the final diagnosis [41]. Of note, the spectrum of diseases underlying non-specific complaints is extremely broad [42], suggesting that copeptin may well be a non-specific marker for disease severity. Copeptin was predictive of 30-day all-cause mortality and elevated levels were associated with an increased mortality in univariate models. Copeptin also provided independent and additional information to clinical risk scores, such as the Katz Index of Independence in Activities of Daily Living and the Charlson Comorbidity Index. Copeptin measurement might therefore facilitate level of care decisions in the ED for this patient group [41].

### Copeptin and other biomarkers

In the ED, copeptin is in competition with other novel biomarkers that are also investigated for their potential use as prognosticators. Apart from established cardiac markers such as BNP, NT-proBNP and derivates of atrial natriuretic peptide [43], other peptides (for example mid-region pro-adrenomedullin [44], ST2 [45] and growth-differentiation factor-15 [46]) are currently the focus of several research groups. The jury is still out on which markers will ultimately make it into clinical routine. The potential advantage of AVP and copeptin over these competing markers in the ED lies in its more central role as one of the key hormones in the body. It is not limited to a single organ system, but is triggered by many disease processes also outside the cardiovascular system. This non-specificity, with respect to a precise diagnostic role, is its strength as a more generalized marker for severe disturbances in patient physiology. In future head-to-head comparisons of biomarkers or biomarker panel strategies copeptin is likely to be one of the strongest candidates.

### Limitations of copeptin

As with most biomarkers there are certain confounding factors for the interpretation of copeptin levels. Copeptin levels were found to be higher in the male volunteers compared with female. Especially in men, there is a strong relationship between copeptin and decreased glomerular filtration rate, probably due to decreased renal copeptin clearance [47]. Furthermore, corticosteroids appear to inhibit copeptin release [48].

## Conclusion

AVP is a substantial part of the endocrine stress response, resulting in ACTH and cortisol release. The trigger for the rapid release of AVP and copeptin in acute disease is not yet clear. Possibly, the body responds to acute and life-threatening diseases by immediate AVP and copeptin release. Copeptin as a biomarker appears to reflect the individual stress level [10].

In the ED, copeptin may be a clinically useful non-specific prognostic marker reflecting disease severity in patients with a wide array of diseases, such as lower respiratory tract infections, heart failure and stroke. Copeptin might assist with risk stratification, resource allocation and disposition planning. However, future studies are needed to evaluate ideal cut-off levels in these clinical scenarios. Additionally, intervention trials are needed to show that determination of copeptin levels in the emergency setting improves patient care.

The concept of a readily available biomarker with the ability to improve prognostic accuracy of known risk stratification tools, such as risk scores, is attractive. Is copeptin, perhaps, the long awaited biomarker that discerns 'sick' from 'not sick'?

## Competing interests

BRAHMS Biomarkers, part of ThermoFisher Scientific, the manufacturer of the copeptin-assay, provided measurements for CHN and RB's study on geriatric emergency patients. No funding was obtained from commercial sources.

## Authors' contributions

CHN, RB and NGM were involved in drafting and editing the manuscript. All authors read and approved the final manuscript.

## Pre-publication history

The pre-publication history for this paper can be accessed here:

http://www.biomedcentral.com/1741-7015/10/7/prepub
